# Catching What We Can't See: Manual Interception of Occluded Fly-Ball Trajectories

**DOI:** 10.1371/journal.pone.0049381

**Published:** 2012-11-14

**Authors:** Gianfranco Bosco, Sergio Delle Monache, Francesco Lacquaniti

**Affiliations:** 1 Department of Systems Medicine, Neuroscience Section, University of Rome Tor Vergata, Rome, Italy; 2 Center of Space Biomedicine, University of Rome Tor Vergata, Rome, Italy; 3 Laboratory of Neuromotor Physiology, Santa Lucia Foundation, Rome, Italy; McMaster University, Canada

## Abstract

Control of interceptive actions may involve fine interplay between feedback-based and predictive mechanisms. These processes rely heavily on target motion information available when the target is visible. However, short-term visual memory signals as well as implicit knowledge about the environment may also contribute to elaborate a predictive representation of the target trajectory, especially when visual feedback is partially unavailable because other objects occlude the visual target. To determine how different processes and information sources are integrated in the control of the interceptive action, we manipulated a computer-generated visual environment representing a baseball game. Twenty-four subjects intercepted fly-ball trajectories by moving a mouse cursor and by indicating the interception with a button press. In two separate sessions, fly-ball trajectories were either fully visible or occluded for 750, 1000 or 1250 ms before ball landing. Natural ball motion was perturbed during the descending trajectory with effects of either weightlessness (0 g) or increased gravity (2 g) at times such that, for occluded trajectories, 500 ms of perturbed motion were visible before ball disappearance. To examine the contribution of previous visual experience with the perturbed trajectories to the interception of invisible targets, the order of visible and occluded sessions was permuted among subjects. Under these experimental conditions, we showed that, with fully visible targets, subjects combined servo-control and predictive strategies. Instead, when intercepting occluded targets, subjects relied mostly on predictive mechanisms based, however, on different type of information depending on previous visual experience. In fact, subjects without prior experience of the perturbed trajectories showed interceptive errors consistent with predictive estimates of the ball trajectory based on a-priori knowledge of gravity. Conversely, the interceptive responses of subjects previously exposed to fully visible trajectories were compatible with the fact that implicit knowledge of the perturbed motion was also taken into account for the extrapolation of occluded trajectories.

## Introduction

Interaction of the motor system with the environment relies on a fine interplay between servo-control processes driven by sensory feedback, and predictive processes, which provide estimates of future states based on prior experience [1]. While sensory feedback may be critical for fine motor control, predictive mechanisms are believed to gain relevance particularly when the action is bound by stringent temporal constraints, because of the delays inherent to the sensory-motor loops [1]–[2]. Catching an object on the fly represents a common exemplification of this type of motor control, since it requires a relatively fast action and accurate estimate of the time and the place for object interception. It has been long debated in the literature whether control of the interceptive action is afforded mainly by servo-mechanisms based on visual feedback or by predictive processes, which provide spatial/temporal estimates of the target interception [3]–[8]. For example, results of psychophysical studies have implied that visual feedback about the target kinematics may underlie moment-to-moment adjustments of the interceptive action [9]–[18]. Along these lines, interceptive strategies based on continuous feedback control have been proposed to explain the ability of baseball players to pursue and catch a fly-ball [19]–[22]. A different approach has been taken by studies inspired by Gibson's ecological theory on visual perception [23], which suggested that optical variables derived directly from object motion information, such as the retinal image dilation rate for objects approaching the observer, could trigger interceptive actions upon reaching threshold values [24]–[31]. However, while the general applicability of this approach has been questioned on several grounds [8], [32]–[33], a growing body of experimental evidence supports the view that time and place of the object interception may be determined predictively also on the basis of prior knowledge [7], [34]–[43]. For example, expectation of target's velocity based on recent history of its kinematics has been shown to influence the kinematics and the timing of the interceptive movement [44]–[46]. A-priori knowledge of invariant features of the environment can also contribute to predictive estimates of the interception timing. Psychophysical and neuroimaging studies have shown, in fact, that manual interception of objects in vertical free-fall reflects temporal predictions based on an internal model of gravity residing in the vestibular cortex [47]–[56]. According to this view, the use of presupposed knowledge of the effect of gravity on the object motion would represent an effective neural strategy to overcome the limited acceleration sensitivity of the visual system [57]–[58]. Predictive processes play undoubtedly a critical role when sensory information about object motion is not available because the object becomes temporarily occluded by other visual elements in the foreground. Despite the momentary absence of visual information, the object may be caught successfully, implying that the central nervous system can predict the time and the place of the interception over the period of visual occlusion [59]–[60]. The ability to perform a successful interceptive action, however, deteriorates remarkably as a function of the visual occlusion interval and with shorter total processing times [61]–[62]. This remarks the complexity of the mechanisms underlying visual motion extrapolation and transformation of the object motion signals into motor commands for the interceptive action. Such processes, in effect, may depend on multiple information sources. For example, information about the target motion kinematics before the occlusion appears to be strongly reflected in the spatial and temporal estimates of manual interception movements [35]–[37], [39], [41], [63]–[65]. These signals are believed to feed a short-term memory representation of the target trajectory, which “fills in” the lack of visual information during the occlusion, providing estimates of the target future positions to guide the interceptive action [66]. Neurophysiological and neuroimaging results support this idea and have identified in parietal area LIP, in cortical premotor areas and in the lateral cerebellum some of the brain regions potentially involved in the predictive representation of the occluded target trajectory [67]–[74].

While integration of visual motion signals available before target occlusion with extra-retinal signals may represent a major component of visual extrapolation, there is also evidence that internalized information, such as the recent history of the target motion, may play a significant role [75]–[76]. Along these lines, psychophysical experiments have suggested short-term adaptations in the motion extrapolation process by observing that early and late occlusion of the visual target may affect differentially the spatial features of catching movements, depending on the presentation order of the occlusion conditions [77]. In addition to short-term mechanisms, abstract long-term categorical representations of the target motion [78], as well as explicit advance information [79] can contribute to this process. Moreover, the evidence that occluding vision for short intervals before interception of vertically free-falling targets with natural and non-natural laws of motion can produce errors consistent with temporal estimates based on an implicit knowledge of the causal effects of Earth's gravity implies a role also for internal models of the external environment [80]. Further support to this idea comes from the recent finding that internal models built through extensive practice with arbitrary accelerations may be taken into account when intercepting occluded visual targets [81].

Within this general framework, the present study sought further insights on the relative contribution of the different processes and information sources to the control of the interceptive action. To this end, we manipulated specific features of a computer-generated visual environment representing a baseball game and asked subjects to intercept simulated fly-ball trajectories (projectile motion), which could be either fully visible or occluded for variable time intervals. Natural ball motion imposed by gravity was altered by introducing the effects of either weightlessness (0 g) or hyper-gravity (2 g) and distinct groups of subjects underwent remarkably different visual exposure to these perturbations, since the order of experimental sessions with either fully visible or occluded trajectories was permuted among subjects. By examining the interceptive errors and the kinematics of the interceptive movements across these experimental conditions, we tried to make inferences on the nature of the underlying control processes. In particular, we tested the possibility that predictive control of the interceptive action, especially in the absence of visual feedback, may rely mainly on spatial/temporal estimates based on a-priori knowledge of Earth's gravity, extending to projectile motion an idea previously dealt with vertical free-falls (see however [82]–[84]). This idea, in effect, would predict a distinctive pattern of interceptive errors signified by systematic temporal and spatial underestimate of 0 g targets and, conversely, by overestimate of 2 g targets. Instead, similar interceptive errors across ball accelerations, coupling between the kinematics of the target and that of the interceptive movement, as well as presence of movement corrections might reveal the contribution of feedback mechanisms. Based on the considerations outlined above, we might expect that implicit knowledge of gravity effects on the target motion would be a primary contributor to the interceptive response, becoming an increasingly stronger factor as longer occlusion intervals reduce the availability of visual information. However, recent findings by de Rugy et al. suggest that novel internal models built from visual experience and practice with arbitrary target trajectories could also influence interception of the occluded targets [81]. We tested this possibility with groups of subjects undergoing remarkably different visual exposure to the perturbed trajectories during the first experimental session. The development of novel visual representations might imply that subjects who acquire visual knowledge of the perturbed trajectories during the first experimental session, even few weeks after the exposure, might exhibit profoundly different patterns of interceptive responses to the occluded targets compared to subjects lacking this visual experience. In particular, if the novel representations interfere with a pre-existing internal model of gravity, we might expect that subjects without full visual experience of the perturbations, by relying primarily on a-priory knowledge of gravity, would intercept the occluded targets with response patterns more congruent with an anticipation of the gravity effects on the target motion.

## Methods

Twenty-four subjects (12 men and 12 women, mean age: 27.1 years±5.6 SD) gave informed written consent to participate in the experimental procedures, which had been previously approved by the ethical committee of the Santa Lucia Foundation. All subjects had normal or corrected-to-normal vision and were right handed or ambidextrous (2 subjects), according to the Edinburgh inventory test. They received modest monetary compensation for their participation. Subjects were seated at a distance of 60 cm from a 21″ computer screen (CRT Philips, model 201 B4), where visual scenes, created with the graphics software Presentation (version 14.4, Neurobehavioral Systems, USA), were displayed with a resolution of 1280×1024, at a refresh rate of 85 Hz. Participants were instructed to keep their head fixed on a chin rest during the experimental trials, but they were allowed to move their eyes freely throughout the visual scene. We recorded the eye movements at a sampling frequency of 250 Hz, by means of the EyeLink II tracker (SR research, CA). However, the analysis of the subjects' oculomotor behavior is not reported here.

### Visual scenes and ball trajectories

**Figure 1:**
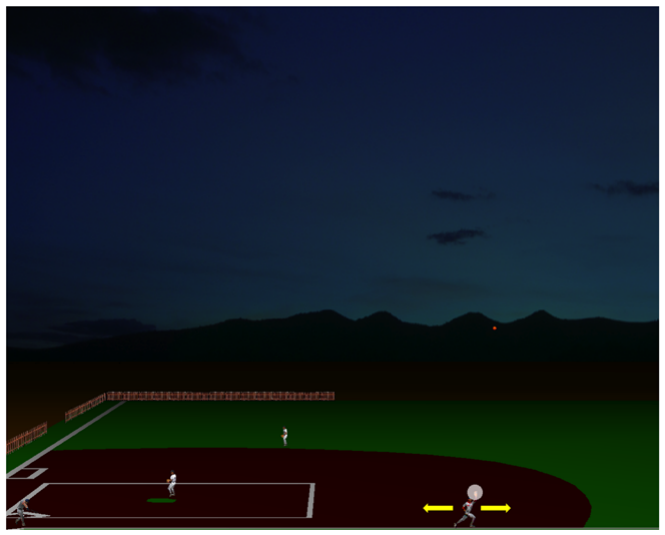
Visual scene displayed during the interception task. The scene represented the fly-ball play of the baseball game. Ball motion (red circle) started from the picture of the batter on the bottom left end of the scene and, by following a parabolic path, landed on the right half of the scene. Note that the animated scene did not reproduce the bat swing at ball launch. In order to intercept the fly-ball trajectory, subjects displaced, with the aid of a computer mouse, the running outfielder either rightward or leftward (yellow arrows indicate possible displacement directions) and pressed the left mouse button to signal the time of interception. The white semitransparent circle around the hand of the outfielder delimited the valid interception zone. In Experiment 2, we provided subjects with knowledge of results by turning the color of the circle either green, to signal successful interception, or red if the ball was missed.

A computer simulation of the baseball action known as the fly-ball was projected on the screen during the experimental trials (Fig. 1). Although the visual scene was designed to provide depth cues through perspective view and relative size, the ball motion (ball diameter: 7 pixels, 0.15° visual angle) was restricted to the frontal plane without apparent displacement in depth. Ball trajectories started, in fact, from the picture of the batter in the bottom left corner of the scene (hitter's bat swing was not rendered, however), followed a parabolic path (or a modified path in perturbed trials, see below) and landed in the bottom right half of the visual scene.

Unperturbed fly-ball trajectories for a projectile, experiencing aerodynamic drag and gravity, were derived from Newtonian mechanics (see [85]):
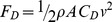
(1)

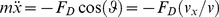
(2)

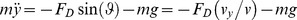
(3)

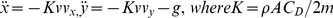
(4)In these equations, *F_D_* indicates the aerodynamic drag force vector magnitude (with its direction being opposite to the velocity vector), 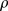 the air density, *A* the ball cross-sectional area, *C_D_* the drag coefficient, *v* the ball speed, i.e. the scalar velocity, with *v_x_* and *v_y_* representing its horizontal and vertical component respectively, *x* ¨ and *ÿ* the horizontal and vertical components of the ball acceleration, *m* the ball mass, 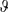 the angle between the ball velocity vector and the horizontal, and *g* the gravity acceleration. Given Eq. 1, we defined the equations of motion for a projectile moving in a two-dimensional field as Eq. 2 and Eq. 3 and obtained Eq. 4 by substituting for *K*. As stated in Brancazio [85], we obtained the *x* and *y* coordinates of the ball by integrating Eq. 4 over time intervals corresponding to each video frame (11.76 ms).

**Figure 2:**
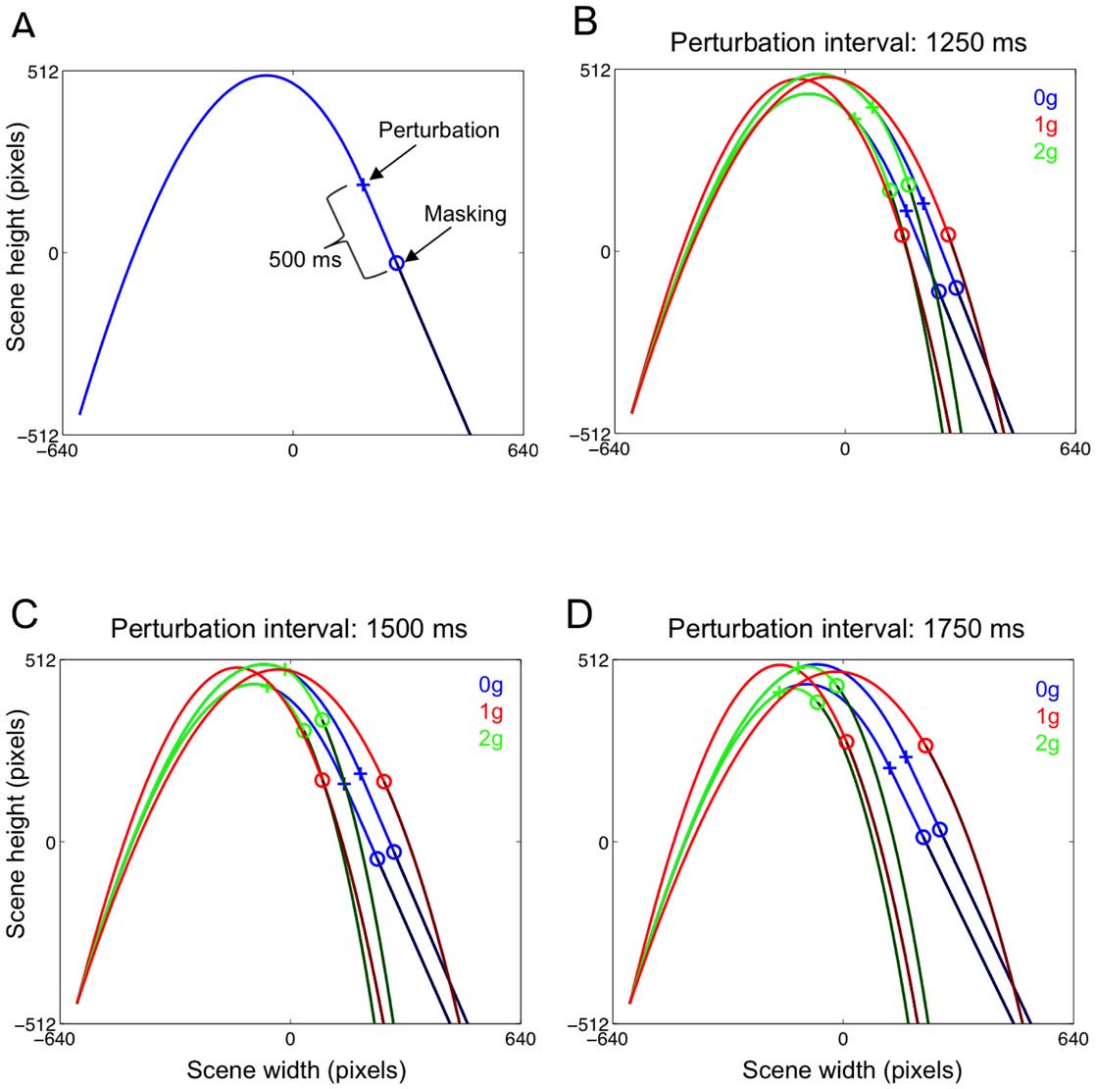
Ball trajectories used during Experiment 1. A. Perturbation and masking onsets are marked with a cross and an open circle, respectively, on an exemplificative 0 g perturbed trajectory. The time interval between the perturbation and the masking event was set to 500 ms. B–D. Each panel illustrates 0 g (blue), 1 g (red) and 2 g (green) trajectories used for each perturbation interval. Note that, for a given perturbation interval, the two corresponding unperturbed 1 g trajectories landed either between 0 g or 2 g perturbed trajectories.

**Figure 3:**
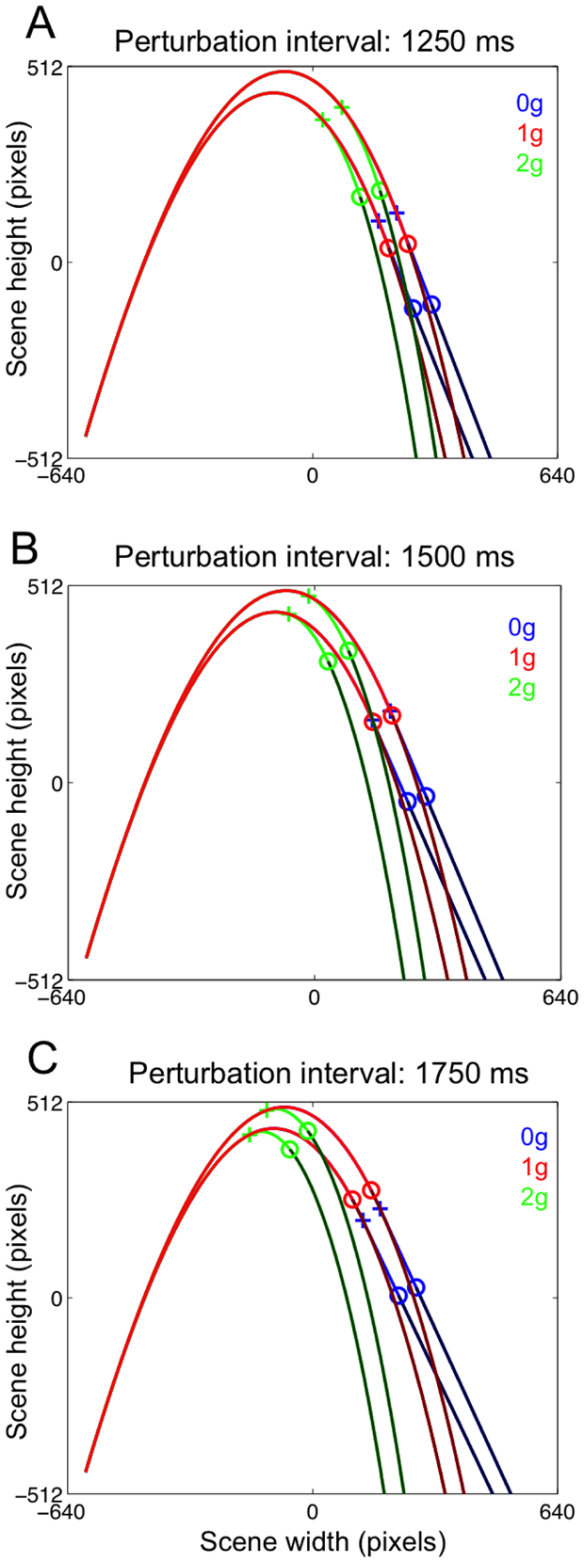
Ball trajectories used during Experiment 2. Each panel illustrates the 0 g (blue), 1 g (red) and 2 g (green) trajectories used for each perturbation interval. Perturbed 0 g and 2 g trajectories were identical to those of Experiment 1. Instead, unperturbed 1 g trajectories had the same initial velocity and launch angle of perturbed trajectories and landed in a separate cluster between the shorter 2 g and the longer 0 g.

We assumed a value of *C_D_* = 0.5 and of *K* = 0.01 m^−1^, corresponding to a ball having a diameter of 10.2 cm and mass of 248 g, slightly bigger and more visible than a regular baseball one. The first portion of the ball trajectories was always unperturbed (governed by the previous equations), whereas the final portion could be perturbed with either 0 g or 2 g laws of motion, or it could retain the natural 1 g law of motion (unperturbed 1 g trials). The trajectory perturbation could occur 1750, 1500 or 1250 ms before the ball reached the interception point. For each perturbation interval, we varied the initial velocity (V_0_) of the ball between two values, 978 and 1016 pixels s^−1^ (corresponding, respectively, to 25.5 and 26.5 m s^−1^ by scaling the scene to real world size), while keeping fixed the launch angle (76.5° relative to the horizontal). In one series of experiments (Experiment 1), unperturbed ball trajectories were designed by varying both the initial direction (either 71.4° or 76.6°) and the ball V_0_ (either 997 pixels s^−1^ corresponding to 26 m s^−1^, or 1016 pixels s^−1^ corresponding to 26.5 m s^−1^), so that half of the unperturbed 1 g trajectories landed close to the perturbed 0 g trajectories and the other half close to the perturbed 2 g trajectories (see Fig. 2B–D). By designing unperturbed trajectories that would land either close to 0 g or to 2 g trajectories, we avoided the possible confound of central tendency effects on the spatial component of the interceptive response to 1 g trials. This design, however, was affected by a potential caveat: because of the different launch angles, unperturbed and perturbed trajectories could be potentially discriminated from the ascending segment of the trajectory. A second series of experiments (Experiment 2) addressed this issue by employing unperturbed 1 g and perturbed (0 g and 2 g) trajectories with the same initial velocities (25.5 and 26.5 m s^−1^) and launch angle (76.5°). Thus, ball trajectories were almost identical until the perturbation, after which they diverged terminating in distinct clusters, with 1 g trajectories landing between the shorter 2 g and the longer 0 g trajectories (Fig. 3). Table 1 summarizes the distribution of ball trajectory durations for the variety of experimental conditions used in the two experiments.

**Table 1:** Ball trajectory durations in ms.

Pert. Length	0 g	1 g (Exp. 1)	1 g (Exp. 2)	2 g
1250	4964.7	4482.4	4341.2	3529.4
1250	5082.4	4435.3	4482.4	3682.4
1000	4823.5	4458.8	4341.2	3764.7
1000	4941.2	4447.1	4482.4	3917.6
750	4682.4	4447.1	4341.2	3952.9
750	4811.8	4458.8	4482.4	4105.9

Trajectory durations are grouped with respect to ball acceleration and ordered according to the length of the perturbation interval and the ball initial velocity.

In order to rule out the possibility that experimental manipulations of the gravity acceleration could be mistaken for 1 g trajectories with altered drag coefficient, we ran simulations where the drag coefficient was either increased five-fold or removed. The resulting trajectories differed remarkably in both spatial extent (mean difference at landing location = 91.8 pixels±31.4 SD) and temporal duration (mean difference = 583.3 ms±170.8 SD) compared to trajectories with altered gravity, ruling out this potential experimental confound.

Each experimental session comprised 10 repetitions, randomly distributed, of the 18 ball trajectories obtained by factoring 2 V_0_ X 3 perturbation intervals X 3 ball accelerations. The 180 trials were delivered in two blocks of 90 trials each, with a 10 minutes pause in between.

Subjects performed two experimental sessions, spaced by about one month apart (mean = 33.25 days±1.6 SD for Experiment 1; mean = 30.7 days±2.7 SD for Experiment 2). In one session, named Visible session, ball trajectories were entirely visible, whereas during the other session the ball disappeared 1250, 1000 or 750 ms before the ball reached the interception height (Masked session). For perturbed trials the interval between the perturbation and the masking event was kept equal to 500 ms by coupling the masking and the perturbation intervals. We randomly assigned participants to the two experiments to groups of 6 subjects each, based on the order of Visible and Masked sessions: Group 1 of Experiment 1 (mean age: 25.5 years±2.3 SD) and Group 3 of Experiment 2 (mean age: 27.3 years±7.2 SD) performed first the Visible session, while Group 2 of Experiment 1 (mean age: 27.3 years±3.8 SD) and Group 4 of Experiment 2 (mean age: 28.5 years±8.4 SD) performed first the Masked session.

Finally, prior to each experimental session, subjects performed a training block of 90 trials in order to familiarize with the interception task. The visual scene and the target motion were different from those presented during the experimental session. At the beginning of the trial, a red rectangular object (24 by 12 pixels, 0.5° visual angle), representing a ball launcher, appeared at the top of the visual scene. The position of the ball launcher along the upper edge of the scene and its orientation relative to the vertical was varied from trial to trial. A ball, same size and appearance as that of the baseball scene, was launched toward the bottom of the visual scene along a rectilinear path determined by the ball launcher orientation. We delivered 5 randomized repetitions of eighteen ball trajectories obtained by varying three positions of the launcher, the motion direction relative to the vertical (three values comprised between −22.5° and 38.6°), and the ball acceleration along its direction of motion (0, 0.6, 1.2 m s^−2^). Overall, these ball trajectories covered the same landing area as the parabolic trajectories used during the experimental sessions.

### Interception Task

Participants used a computer gaming mouse (Razer copperhead, Razer USA) to displace the image of the running outfielder along the horizontal axis from its initial center position to the optimal position for intercepting the ball (see Figure 1). A white semitransparent circle centered on the outfielder's hand (diameter: 28 pixels, 0.32° visual angle) delimited the valid interception area and subjects were instructed to indicate the time of the interception by pressing the left mouse button. In Experiment 1, we did not provide feedback of the interception response and, thus, the task was performed open-loop. Instead, in Experiment 2 we provided visual feedback of the interception error: the white semi-transparent circle turned green if the ball was intercepted successfully, otherwise it turned red and the ball exploded, remaining visible for 200 ms at the location reached at the time of the button press. With Experiment 2 we could, then, determine whether the interceptive responses to the visible and the occluded trajectories were adapted with practice.

**Figure 4:**
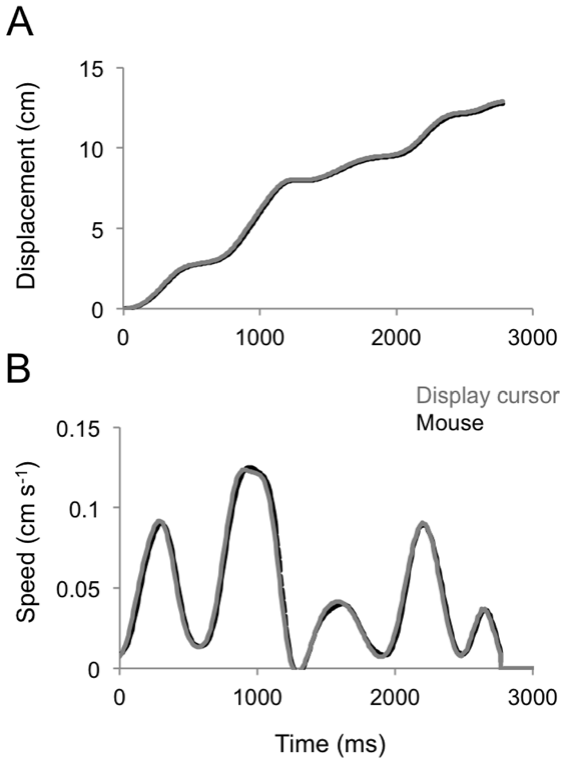
Relationship between mouse and display cursor displacements. Mouse movements recorded with a motion capture system (black trace) are compared with display cursor movements recorded by means of the software Presentation (grey trace) during a typical interception trial. A. Mouse displacement. B. Mouse speed.

With regard to the relationship between mouse displacements and movements of the running outfielder, we set to unity their ratio by adjusting empirically the mouse sensitivity within the software platform Presentation. In three separate experiments, we measured this ratio across the range of mouse movements performed during the interception task in order to rule out possible movement-dependent distortions. To this end, we acquired the position of a reflective marker placed on the mouse surface by means of a motion capture system (Smart 100DX, BTS; sampling frequency: 100 Hz) and computed the mouse to display cursor displacement ratio every 200 ms from the onset to the end of the movement. The value of the ratio was, on average, equal to 0.99±0.02 SD, without evident distortions across the range of movement speeds typically observed during the interception task (see the example in Figure 4).

After each interception trial, subjects returned the outfielder at its home position located at the center of the scene. In order to facilitate this procedure, a yellow circle (diameter: 30 pixels, 0.32° visual angle) appeared at the current position of the outfielder, while a red ring (inner diameter: 45 pixels, 0.47° visual angle) indicated the initial home position. The color of the ring turned to green when the yellow circle entered the ring, and the next trial was allowed to begin if the yellow circle was kept inside the ring for at least 500 ms.

### Data Acquisition

Mouse button presses were acquired at a sampling frequency of 1 KHz through a PC-based data acquisition interface (CED Power 1401). The x-y positions of the mouse cursor were sampled at a nominal frequency of 170 Hz through the USB communication protocol available in the software platform Presentation (Neurobehavioral Systems, USA). Mouse position samples were time-locked to the video frames: one sample was acquired at the video frame onset and a second one after 6 ms. Mouse cursor horizontal position signals were used for real-time rendering of the outfielder's displacements, as well as for off-line analyses (see below).

### Data Analysis

#### Button press responses

We considered three measures of interception error: the timing error (TE), the position error (PE) and the scalar error vector (ME). TE was defined as the difference between the button press time and the time at which the ball trajectory intersected the interception point. Positive and negative TEs denoted late and early responses, respectively. PE was the difference in centimeters between the horizontal position of the mouse cursor at the time of the button press and the interception point. Negative values indicated horizontal underestimate of the landing position of the ball, while positive values indicated overestimate. ME was the modulus of the spatial error vector, that is, the Euclidean distance in centimeters between the mouse cursor and the ball (both horizontal and vertical coordinates) at the time of the button press.

Since the direction of the errors in both temporal and spatial domain could be indicative of the underlying interceptive processes, TE and PE were used as main measures of the interceptive error to evaluate how the subjects' performance varied in relation to the experimental manipulations of the ball trajectory. Thus, we computed mean values of TE and PE across repetitions of each experimental condition and carried out repeated measures mixed-ANOVAs with ball acceleration, ball initial velocity (V_0_) and the length of the perturbation interval as within subject factors, and session order as between subject factor. The cut-off for statistical significance of the ANOVA effects to was set to p = 0.05 (Greenhouse-Geisser corrected) and Bonferroni post-hoc analyses were performed on the significant effects of the repeated measures ANOVA. Separate ANOVAs were run for data of the two experiments and of the Visible and Masked sessions. In addition, we used one sample t-tests in order to determine whether TE and PE distributions deviated significantly from the correct response (TE = 0 and PE = 0).

For Experiment 2, we analyzed also the scalar component of the error vector (ME) because it was directly related to the response feedback provided during this experiment. We evaluated how the ME varied as a function of the experimental conditions by computing mean values across repetitions of each experimental condition and by performing repeated measures mixed-ANOVAs with ball acceleration, ball initial velocity (V_0_) and the length of the perturbation interval as within subject factors, and session order as between subject factor. In addition, we assessed the effect of practice on the interceptive responses, by examining the time course of ME with successive trials of the same law of motion. Briefly, we computed for each trial the mean ME across subjects belonging to each experimental group, and fitted separate exponential curves for 0 g, 1 g and 2 g responses, according to the following equation:
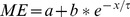
where *x* represents the series of 60 trials of each law of motion and τthe time constant of the exponential change of the scalar error along the trial series.

#### Mouse cursor movements

**Figure 5:**
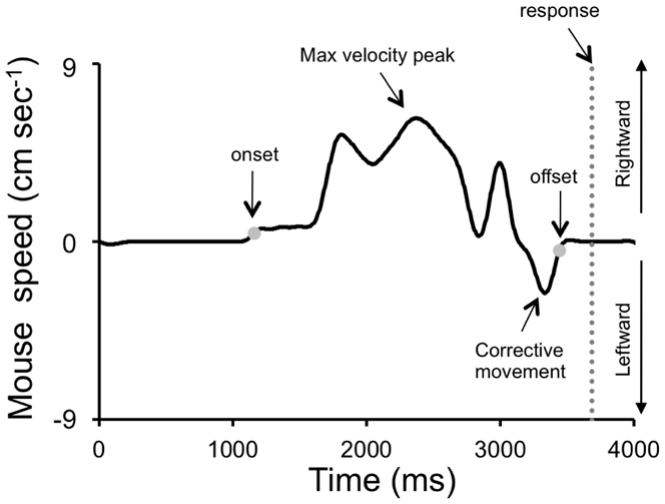
Mouse velocity profile and indexes. A typical multi-peaked mouse velocity trace recorded from one subject during the Visible session. Positive and negative mouse velocities correspond to rightward and leftward outfielder displacements, respectively. Black arrows indicate mouse velocity profile events from which we derived the indexes considered for further statistical analyses. The grey dotted line indicates the button press response time.

The time-series of the horizontal position of the mouse cursor were differentiated and filtered with a zero-lag 2^nd^ order Butterworth filter (cut-off frequency: 5 Hz). The resulting velocity profiles showed several peaks, indicating multiple movement sub-components (Figures 4 and 5). Positive peaks denoted rightward displacements of the outfielder toward the interception area, whereas negative peaks corresponded to leftward movements of the outfielder back to the center position. These latter occurred usually near the end of the trial, often just before the button press (see example in Fig. 5), perhaps representing the subjects' final attempts to correct the position of the outfielder before the interception response [38], [77].

In order to provide a synthetic description of the mouse cursor kinematics, we considered several indexes derived from the velocity profile. The movement onset was defined as the time-point when the mouse velocity first exceeded 0.4 cm s^−1^ and, by the same criterion, the end of the movement was the time-point when the mouse velocity decreased below 0.4 cm s^−1^ after the last detectable local maximum. Movement duration was the time interval comprised between these two events. Other indexes we considered for further statistical analyses were: the latency and the value of the highest velocity peak, the direction of the last peak before the button press response and the mouse velocity at the time of the button press (see Figure 5).

We used multivariate analyses to relate each index to the ball trajectory, the session type and the session order. In particular, repeated measures mixed-ANOVAs with the ball trajectory (18-level categorical variable) and the session type as within subject factors and the session order as between subjects factor were used to analyze movement onset, movement duration, latency and value of the maximal peak velocity. In these ANOVA models, we did not considered explicitly the ball kinematics because movement onsets and maximal velocity peaks occurred usually before the perturbation, the main factor determining the ball trajectory kinematics.

The effect of the ball acceleration was modeled explicitly on the direction of the last velocity peak and the mouse velocity at button press, as these variables were sampled after the trajectory perturbation. Note, however, that data from Experiment 2 could not be used for this analysis because ball accelerations and trajectory spatial lengths were univocally related (see Figure 3), creating a potential experimental confound. Instead, by designing 1 g trajectories that landed close to either 0 g or 2 g trajectories, Experiment 1 was not affected by this confound (see Figure 2). We used General Linear Model (GLM) to relate the mouse velocity at button press to the ball acceleration, the session type, the session order and all the interactions between these factors. To analyze the direction of the last velocity peak, which was a binary variable, we applied a logistic regression with the same model predictors used in the GLM. In order to simplify the interpretation of both these regression analyses, we also performed backward iterative elimination of the least significant predictors by setting the significance cut-off to α = 0.05.

## Results

### Button press responses

We focused mainly on timing (TE) and position errors (PE), because their distributions, by indicating either under- or over-estimate of the ball trajectories, could be suggestive of an underlying interceptive strategy. For example, prediction of ball motion based on a-priori knowledge of Earth's gravity might imply distributions of TE and PE for the perturbed trajectories characterized by temporal and spatial underestimates of 0 g targets and overestimates of 2 g targets. Conversely, similar interceptive errors across ball accelerations might denote a strategy based on visual feedback of the ball motion, like that proposed for the prospective strategy. We also considered the possibility that, when intercepting occluded targets, previous visual experience of the perturbed trajectories might contribute to the visual extrapolation process at the expense of pre-conceived knowledge of the effect of gravity on the ball motion. This may imply: 1) distributions of TE and PE more consistent with the pattern predicted by the internal model of gravity in subject groups who underwent first the Masked session compared to those performing first the Visible session; 2) adaptation of the interceptive responses to the altered trajectories during the Visible session.

#### Visible Session

**Figure 6:**
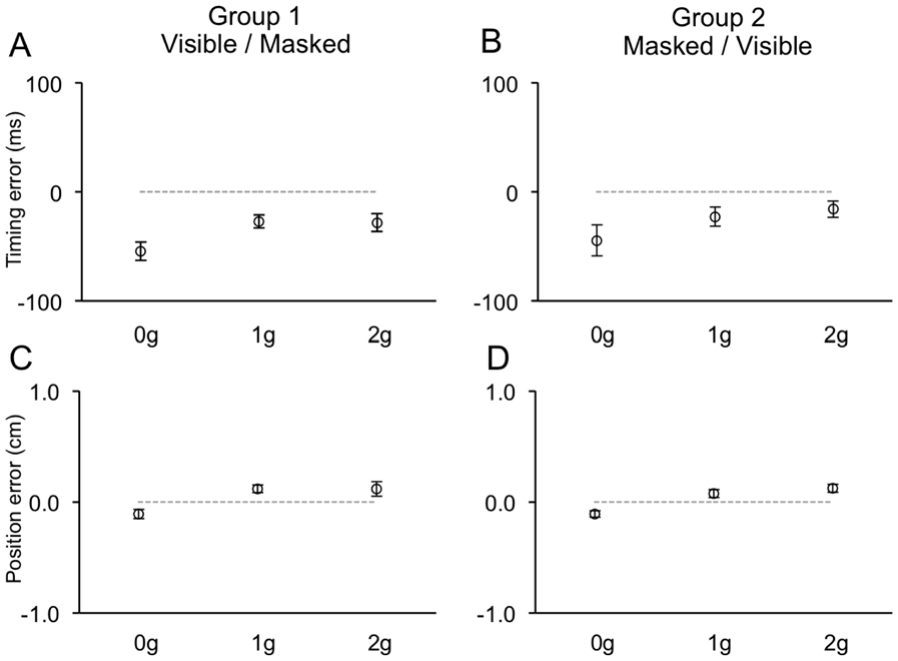
Distributions of timing (A–B) and positional errors (C–D) recorded during the Visible session of Experiment 1. Data-points represent average values (± SEM) computed for each ball acceleration among subjects belonging to either Group 1 (left column) or Group 2 (right column).

**Figure 7:**
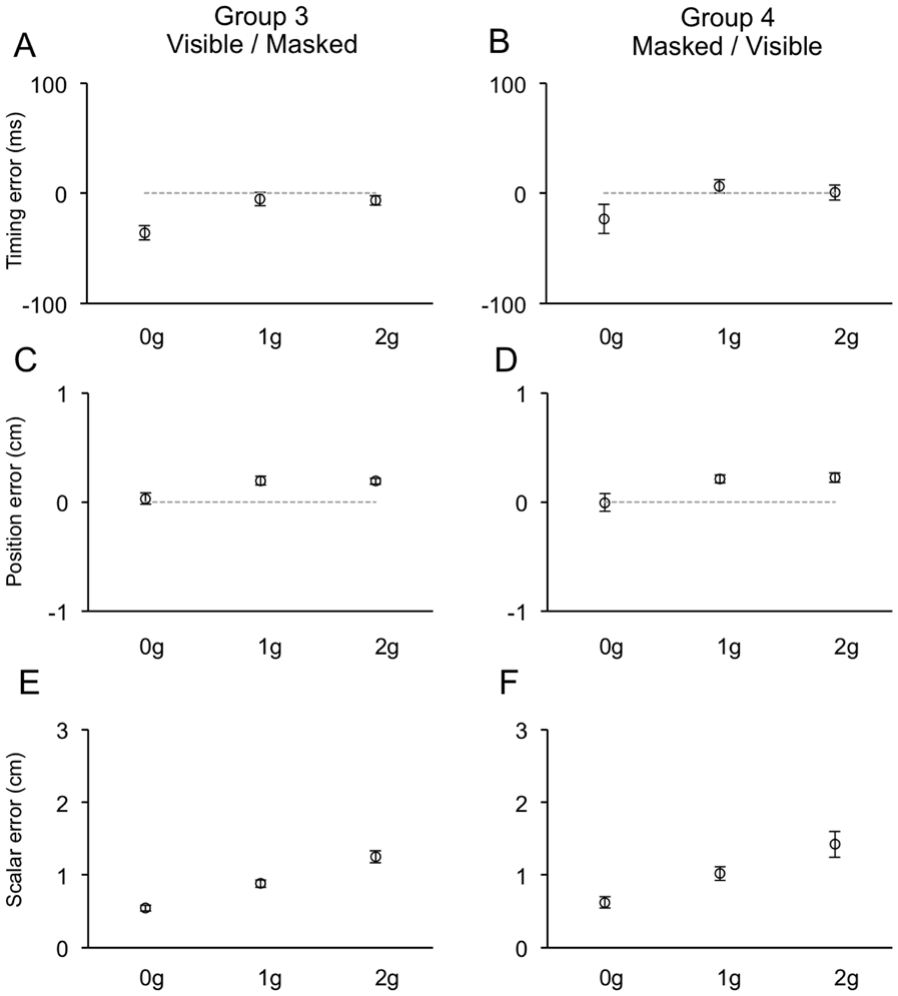
Distributions of timing (A–B), positional (C–D) and scalar (E–F) errors recorded during the Visible session of Experiment 2. Same layout as Figure 6.

When the ball was visible throughout the trajectory, participants made rather small temporal and positional errors. Repeated measures ANOVAs showed that, in both experiments, the timing of the interceptive response depended significantly only on the ball acceleration (F_(2, 20)_ = 11.2, p = 0.006; F_(2, 20)_ = 14.2, p = 0.001 for Experiment 1 and 2, respectively). These effects were explained by the earlier responses observed, on average, for 0 g perturbed trajectories compared to 1 g and 2 g trajectories, which produced similar errors. During Experiment 1, interceptive responses were slightly early also for accelerated 1 g and 2 g trials, whereas during Experiment 2, both 1 g and 2 g trials were timed correctly (see Table 2). Response timing, however, did not depend significantly on the order of the experimental sessions, as indicated by the similar TE distributions between subject groups performing first either the Visible or the Masked session (compare right and left column graphs in Figures 6 and 7).

**Table 2:** Mean (± SEM) timing and position errors recorded during the Visible session of Experiment 1 and Experiment 2 (n = 12 for both experiments).

		TE (ms)		PE (cm)	
	*Acceleration*	*Mean*	*SEM*	*Mean*	*SEM*
***Experiment 1***	***0 g***	−49.5	8.0	−0.11	0.02
	***1 g***	−24.9	5.1	0.1	0.02
	***2 g***	−22.1	5.6	0.12	0.03
***Experiment 2***	***0 g***	−29.5	7.3	0.01	0.04
	***1 g***	0.4	4.4	0.2	0.03
	***2 g***	−2.8	4.0	0.2	0.02

Position errors, albeit very small, depended also on the ball acceleration (F_(2, 20)_ = 39.8, p<0.001; F_(2, 20)_ = 23.0, p<0.001 for Experiment 1 and 2, respectively). These effects were explained by a different response behavior to accelerated 1 g and 2 g trajectories compared to 0 g trajectories. In Experiment 1, both 1 g and 2 g trajectories were slightly overestimated, whereas 0 g trajectories were underestimated. During Experiment 2, mouse positioning was, on average, correct for 0 g trajectories, while accelerated trajectories were slightly overestimated (see Table 2). Like the response timing, the mouse positioning did not depend significantly on the experimental session order (compare left and right columns in Figures 6 and 7). Overall, the distributions of temporal and spatial errors during the Visible session may suggest the integration of predictive and feedback-based mechanisms. Although the consistently early response timing to 0 g perturbed trajectories might be compatible with presupposed knowledge of Earth's gravity effects on the ball motion, the similar temporal and spatial responses between accelerated 1 g and 2 g trials and the correct mouse positioning in response to 0 g motion might indicate that these predictive estimates could be overridden by servo-controlled processes based on visual feedback. Evidence in favor of this interpretation emerged also by the analysis of the mouse cursor kinematics (see below).

For the dataset collected during Experiment 2, we also examined the ME distributions across experimental conditions, since ME represented a measure of the interceptive error related more directly to the response feedback provided in this experiment (see methods). We found that ME depended significantly only on the ball acceleration (F_(2, 20)_ = 51.9, p<0.001), with a monotonic increase from 0 g to 2 g trials that was equally evident in both Group 3 and Group 4 subjects (Figure 7E–F). This monotonic increase of the interceptive error with ball acceleration, however, may simply reflect the higher terminal velocities of accelerated targets and, thus, the shorter times available for their interception.

#### Masked session

**Figure 8:**
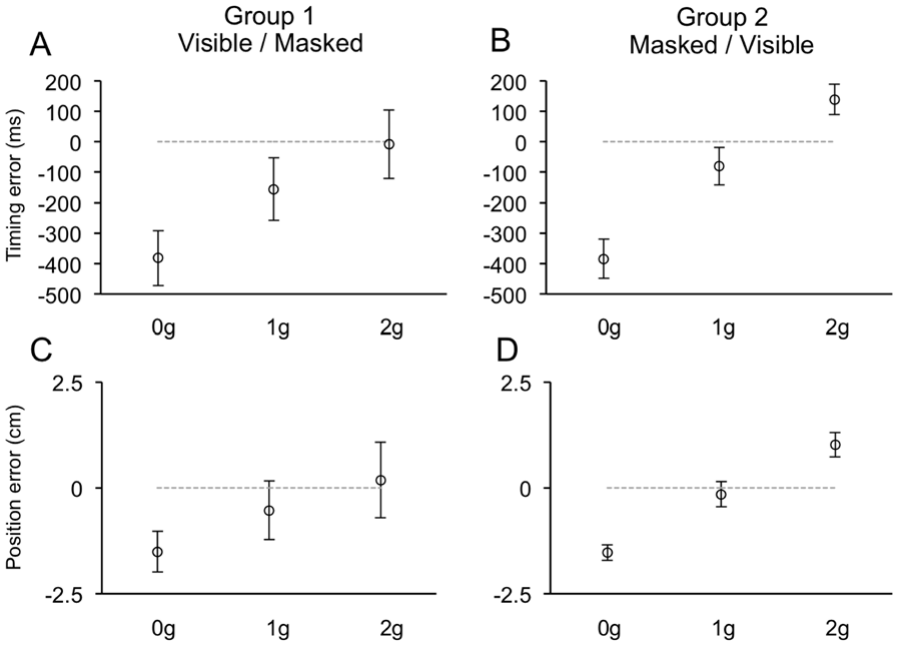
Distributions of timing (A–B) and positional (C–D) errors recorded during the Masked session of Experiment 1. Same layout as Figure 6.

**Figure 9:**
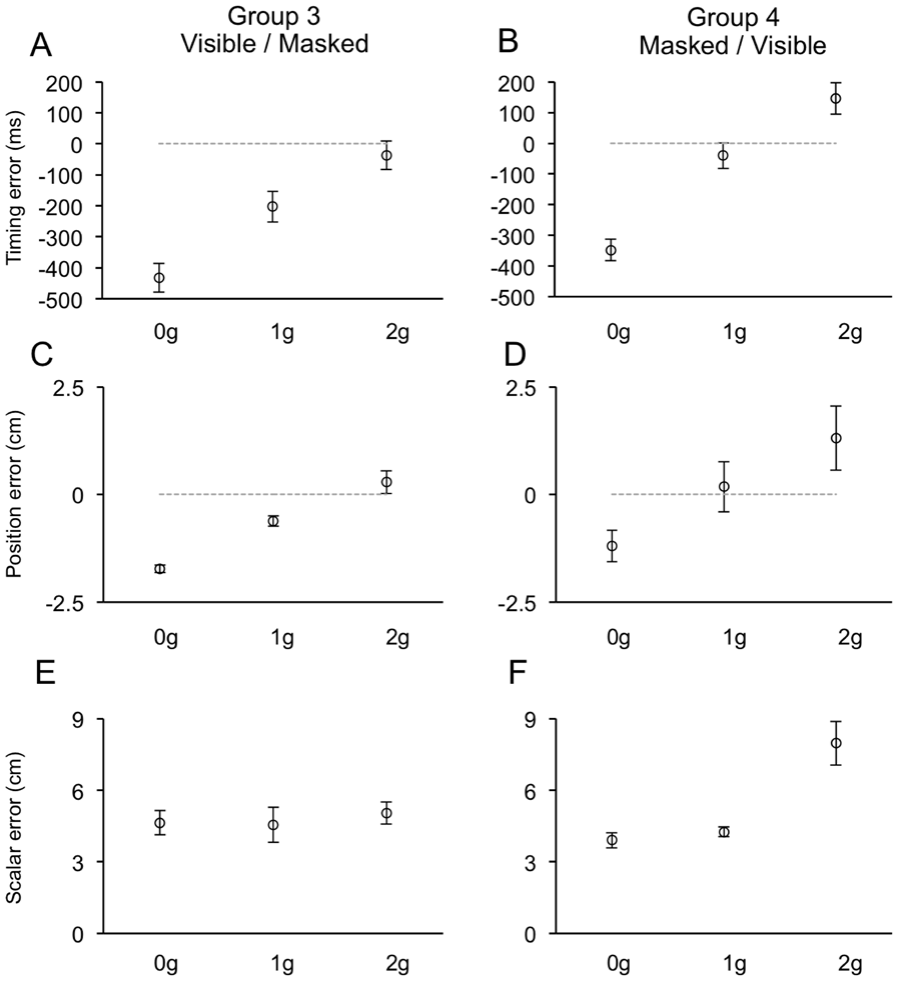
Distributions of timing (A–B), positional (C–D) and scalar (E–F) errors recorded during the Masked session of Experiment 2. Same layout as Figure 6.

When visual targets were occluded, subjects made rather large temporal and positional errors, which followed monotonic trends with respect to the ball acceleration (see Table 3). Ball acceleration, in fact, represented the strongest factor explaining the variance in both TE (F_(2, 20)_ = 198.8, p<0.001; F_(2, 20)_ = 416.2, p<0.001 for Experiment 1 and 2, respectively) and PE (F_(2, 20)_ = 69.0, p<0.001; F_(2, 20)_ = 70.3, p = 0.001 for Experiment 1 and 2, respectively). Responses to perturbed trajectories followed a general pattern consistent with a-priori assumptions of the effect of Earth's gravity on the ball motion. In fact, 0 g trajectories were systematically underestimated, both with respect to the timing and the position for ball interception, whereas 2 g trajectories were overestimated. However, responses to 1 g trajectories were systematically early and denoted slight spatial underestimate of the target trajectory. Thus, although in both experiments the distributions of TE and PE across ball accelerations could be consistent with implicit knowledge of gravity effects, some inconsistency emerged with respect to the responses to 1 g trajectories. This inconsistency could be explained, for most part, by a different response behavior between subject groups who performed the Masked session either before or after the Visible session. In both experiments, in fact, a significant fraction of the variance in the TE was accounted for by first order interaction effects of ball acceleration and session order (F_(2, 20)_ = 5.5, p = 0.038; F_(2, 20)_ = 6.3, p = 0.01 for Experiment 1 and 2, respectively). Group 2 and Group 4 subjects, who performed the Masked session first, showed very early responses to 0 g trajectories, late responses to 2 g trajectories and responses to 1 g trajectories very close to the correct timing, like predicted by the internal model of gravity. Instead, Group 1 and Group 3 subjects, who had previous full visual experience of the perturbed trajectories, showed very early responses to 0 g trajectories (undistinguishable from those of the other two groups; p>0.05 two sample t-test) and to 1 g trajectories, but almost correct timing in response to perturbed 2 g trajectories (Figures 8–9). Position errors followed a similar pattern. Group 2 and Group 4 subjects estimated correctly the unperturbed 1 g trajectories (p>0.05, one sample t-test) while they grossly underestimated the 0 g trajectories and overestimated the 2 g trajectories (p>0.05, one sample t-test). Group 1 and Group 3 subjects underestimated significantly not only the 0 g trajectories (by the same amount as Group 2 and 4 p>0.05, two sample t-tests), but also the unperturbed 1 g trajectories (p<0.05, one sample t-tests). Instead, their PE were closest to null (p>0.05) in response to the perturbed 2 g trajectories. Despite the similarities with the TE, interaction effects between ball acceleration and session order did not, however, account for a statistically significant fraction of the PE variance.

**Table 3:** Mean (± SEM) timing and position errors recorded during the Masked session of Experiment 1 and Experiment 2 (n = 12 for both experiments).

		TE (ms)		PE (cm)	
	*Acceleration*	*Mean*	*SEM*	*Mean*	*SEM*
***Experiment 1***	***0 g***	−383.2	52.9	−1.5	0.2
	***1 g***	−118.1	58.4	−0.3	0.4
	***2 g***	65.1	62.9	0.6	0.4
***Experiment 2***	***0 g***	−390.1	30.4	−1.5	0.2
	***1 g***	−121.3	39.5	−0.2	0.3
	***2 g***	54.6	43.0	0.8	0.4

Both temporal and spatial errors became significantly larger as a function of the occlusion interval (TE: F_(2, 20)_ = 22.4, p<0.001 for Experiment 1 and F_(2, 20)_ = 84.7, p<0.001 for Experiment 2; PE: F_(2, 20)_ = 5.4, p = 0.03 and F_(2, 20)_ = 14.8, p = 0.002 for Experiment 1 and 2, respectively). Moreover, the dependence of the interceptive response on the ball acceleration was greatly influenced by the length of the occlusion, as TE and PE distributions among ball accelerations were more separated at longer occlusion intervals. Highly significant two-way interaction effects between the length of the masking interval and the ball acceleration accounted for this response pattern (TE: F_(4, 40)_ = 30.7, p<0.001 for Experiment 1 and F_(4, 40)_ = 18.8, p<0.001 for Experiment 2; PE: F_(4, 40)_ = 20.8, p<0.001 for Experiment 1 and F_(4, 40)_ = 28.1, p<0.001 for Experiment 2). Finally, during Experiment 2, we found small, albeit statistically significant, effects of the ball initial velocity (F_(1, 10)_ = 7.4, p = 0.02) as well as of the interaction between the ball initial velocity and the session order on the mouse cursor positioning (F_(1, 10)_ = 5.3, p = 0.04).

Alike the Visible session, the distribution of ME observed in Experiment 2 was significantly dependent on the ball acceleration (F_(2, 20)_ = 11.8, p = 0.002). However, this effect was accounted for by larger scalar errors during 2 g trials (mean ME = 6.5 cm±0.6 SEM), compared to 0 g (mean ME = 4.3 cm±0.3 SEM) and 1 g trials (mean ME = 4.4 cm±0.4 SEM), which were similar. The similar error size for 0 g and 1 g trajectories could not be expected from the distribution of ball terminal velocities, and, perhaps, may represent additional evidence in favor of an implicit knowledge of Earth's gravity. Interestingly, this general pattern was mainly determined by the responses of Group 4 subjects, whereas Group 3 subjects showed almost equal scalar errors among ball accelerations (Figure 9E–F). A significant interaction effect between ball acceleration and session order accounted for this observation (F_(2, 20)_ = 8.1, p = 0.01). Finally, like noted above for TE and PE, scalar errors were significantly larger with longer occlusion intervals (F_(2, 20)_ = 36.5, p<0.001).

Overall, these results are compatible with the idea that when intercepting occluded targets, subjects without prior full visual experience of the ball trajectories (Group 2 and Group 4) relied mainly on presupposed knowledge of the effects of gravity on the motion of the visual target. Instead, Group 1 and Group 3 subjects, who performed first the Visible session, extrapolated the masked trajectory by using a weighted combination of prior visual knowledge of the ball trajectories and internal knowledge of the effects of gravity.

#### Adaptation of interceptive responses

**Figure 10:**
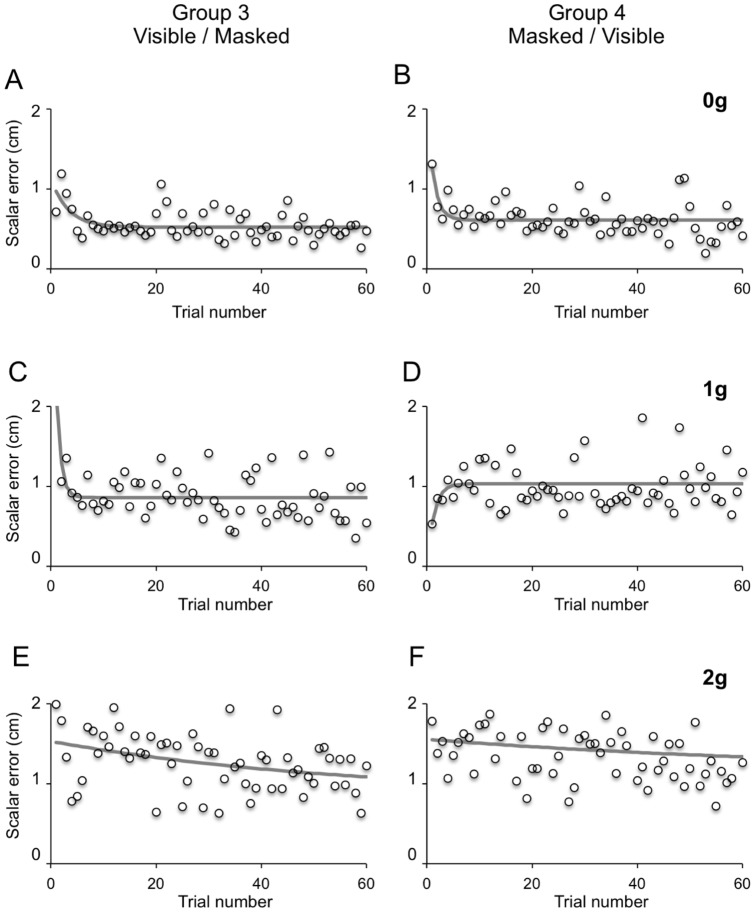
Adaptation of button press responses. Time series of ME along successive 0 g (A–B), 1 g (C–D) and 2 g (E–F) trials of the Visible session of Experiment 2. Each data point represent the mean value among subjects belonging to either Group 3 (left column) or Group 4 (right column). Grey solid lines represent best exponential fits to the time series.

The finding that prior visual experience of the perturbed trajectories influenced the interceptive responses to occluded targets may imply that visual memory, acquired during the Visible session, was retained at least until the Masked session performed about one month later. In Experiment 2, this possibility could be tested explicitly because subjects received visual feedback of the interceptive error and, thus, could adapt their responses by trial and error. During the Visible session, adaptation of the interception responses was evident for all ball accelerations, with the exception of 2 g trials in Group 4 subjects (Figure 10). Adaptation to both 0 g and 1 g trajectories occurred within few trials. In fact, the time series of ME were best fit by single exponential curves with time constants ranging from 0.9 to 3.2 trials. Adaptation of the interceptive responses of Group 3 subjects to 2 g trajectories occurred, however, with a much longer time constant of 57.4 trials. Interestingly, Group 3 and Group 4 showed opposite adaptation trends to 1 g trials. In fact, while Group 3 showed a rapid decrease of the ME, in Group 4 the interception performance degraded slightly during the first couple of trials before reaching a stable level. In all cases, ME values at the curve asymptote were above the threshold for a successful response, indicating incomplete adaptation and rather modest overall success rates. Group 3 subjects intercepted successfully 145/357 (40.6%) 0 g trials, 85/358 (23.74%) 1 g trials and 53/355 (14.9%) 2 g trials, whereas Group 4 subjects intercepted a slightly lower fraction of trials that is, 131/344 (38.08%) 0 g trials, 59/341 (17.3%) 1 g trials and 40/351 (11.4%) 2 g trials. During the Masked session, both groups of subjects did not show adaptation of the button press responses. In fact, none of the ME time series could be fit well by exponential curves. This variant of the task was very challenging to participants and resulted into very low interception rates. Group 3 subjects intercepted only 2/360 0 g trials (0.6%), 3/357 1 g trials (0.8%), 9/353 2 g trials (2.54%) and Group 4 intercepted 1/358 (0.3%) 0 g trials, 3/349 (0.8%) 1 g trials and 3/319 (0.9%) 2 g trials.

We also analyzed the Experiment 1 dataset, even though subjects did not have knowledge of results and adaptation of interceptive responses was not an expected result. Instead, even without response error feedback, both Group 1 and Group 2 subjects adapted the interceptive responses to 0 g and 2 g trajectories during the Visible session. Learning rates were comparable to those observed in Experiment 2, with time constants ranging from 2.4 to 6.1 trials. Responses to 1 g trajectories were not adapted, however. Success rates were also the same order of magnitude as those reported for Experiment 2. Group 1 subjects intercepted successfully 131/356 (36.8%) 0 g trials, 98/358 (27.3%) 1 g trials and 58/360 (16.1%) 2 g trials, whereas Group 2 subjects intercepted a slightly higher percentage of trials namely, 177/358 (49.44%) 0 g trials, 107/359 (29.8%) 1 g trials and 80/360 (22.2%) 2 g trials. Like in Experiment 2, responses to occluded trajectories were not adapted and, in fact, success rates were also extremely low. Group 1 subjects intercepted only 1/359 0 g trials (0.28%), 1/348 1 g trials (0.29%), 2/319 2 g trials (0.62%) and Group 2 intercepted 1/360 (0.28%) 0 g trials, 9/357 (2.5%) 1 g trials and 0/327 (0%) 2 g trials.

### Mouse cursor movements

#### General features

**Figure 11:**
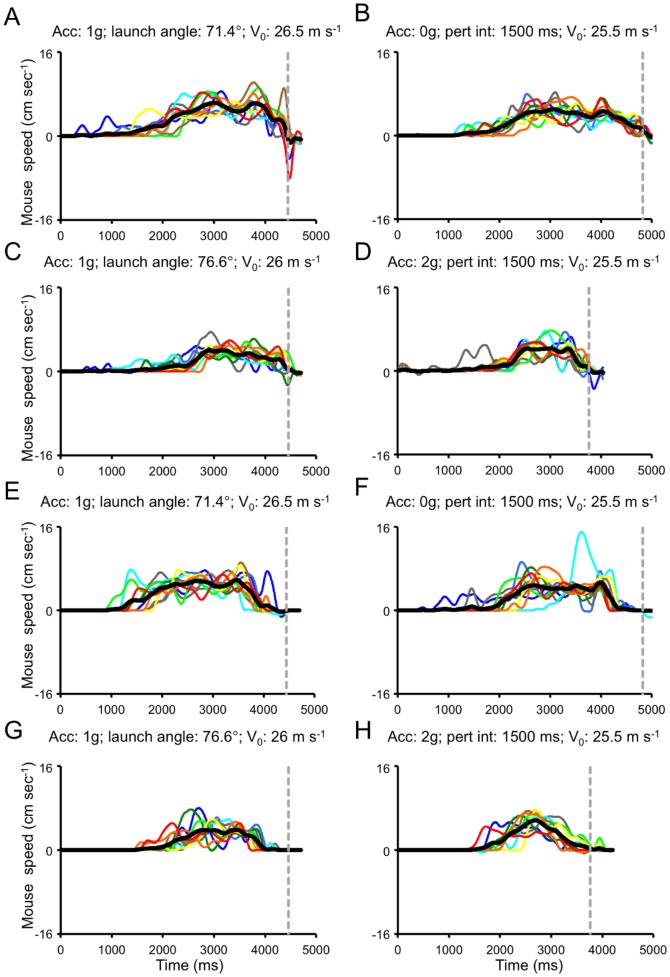
Mouse movements recorded from an exemplificative subject belonging to Group 1 of Experiment 1. Each panel shows all the valid trials recorded for a given experimental condition. Successive repetitions are color-coded from cold to warm hues, while the thick black line represents the mean among repetitions. The grey dashed line indicates the time of the button press response. Left panels illustrate mouse movements following unperturbed 1 g trajectories matched for spatial length with the perturbed conditions in the right panels. A–D. Visible session. E–H. Masked Session.

**Figure 12:**
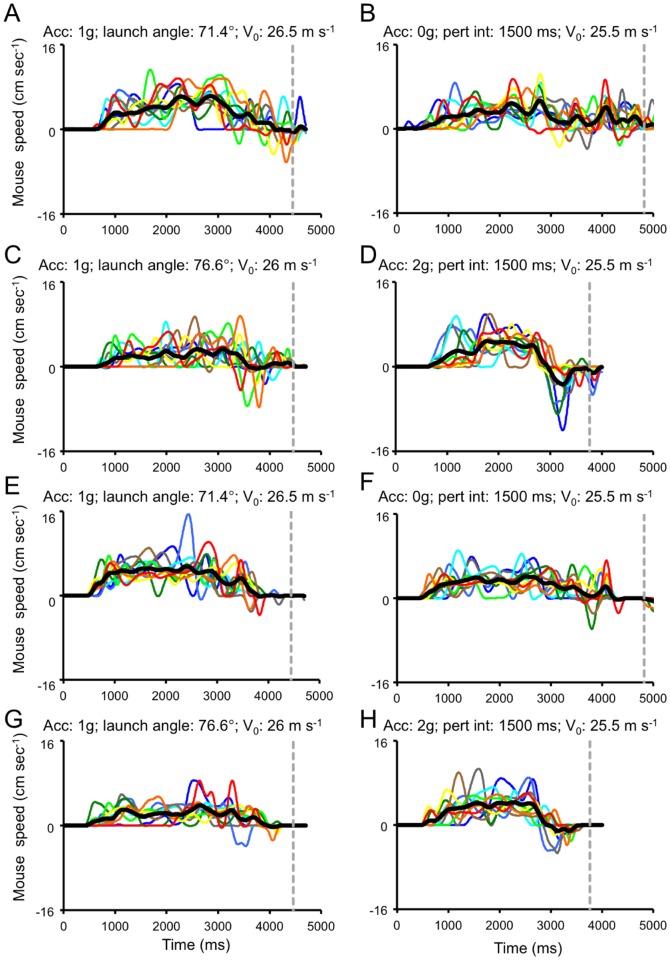
Mouse movements recorded from an exemplificative subject belonging to Group 2 of Experiment 1. Same layout as Figure 8.

Prototypical examples of mouse velocity profiles for 8 ball trajectories recorded during the Visible (panels A–D) and the Masked (panels E–H) sessions are illustrated for two representative subjects in Figures 11 (MiRu, Group 1) and 12 (FrDi, Group 2). Mouse velocity profiles generally featured multiple peaks, denoting distinct movement subcomponents. In this regard, movement velocity profiles were similar to those described previously by Lee et al. [86] for manual interception of visual targets moving at slow speeds along circular paths. Subjects initiated, usually, the mouse movement just before the ball trajectory reached the apex, and displaced the mouse toward the interception area by making several sub-movements. They tended to reach the desired position for interception well before the arrival of the visual target, while performing small corrections of the mouse position around the time of the button press response. In line with these qualitative observations, we found that in the majority of trials (59.9%, 2553/4258 in the Visible session and 78.6%, 3279/4166 in the Masked session) the mouse cursor speed at the time of the button press response was below 1.4 cm s^−1^ and it was below the 0.4 cm s^−1^ threshold in a considerable number of cases (33.3% and 51.8% in the Visible and Masked session, respectively). Finally, subjects did not necessarily adopt a stereotypical kinematic profile across repetitions of the same experimental condition, even though the final mouse cursor position was invariably reached well before the arrival of the visual target (see, for example, the large inter-trial variability shown by the subject illustrated in Fig. 12).

#### Multivariate analyses of mouse cursor kinematics

**Figure 13:**
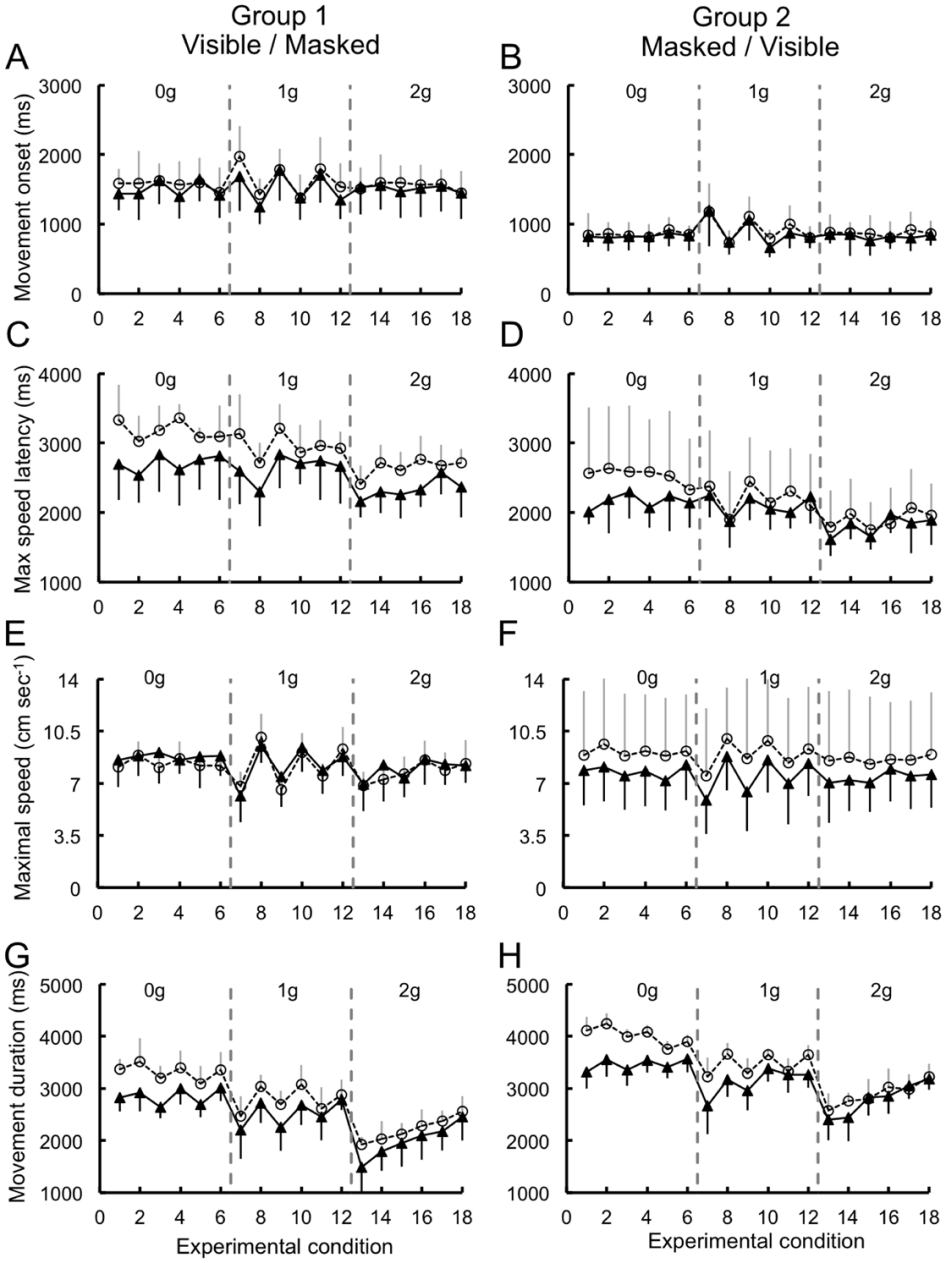
Indexes derived from mouse velocity profiles recorded during Experiment 1. Each data point represents the mean value (± SEM) computed for each experimental condition among subjects belonging to either Group 1 (right column) or Group 2 (left column). Open circles, Visible session; Filled back triangles, Masked session. Vertical dashed grey lines separate experimental conditions with different laws of motion (1–6: 0 g; 7–12: 1 g; 13–18: 2 g). A–B. Movement onset. C–D. Latency of the maximal velocity peak. E–F. Maximal mouse velocity. G–H. Movement duration.

To determine how mouse cursor kinematics varied in relation to the experimental manipulations, we analyzed the following indexes: movement onset, latency of the maximal peak velocity, maximal peak velocity, movement duration, mouse velocity at the time of the button press response and the direction of the last sub-movement before the button press. Repeated measure ANOVAs on the first four of these indexes provided a quantitative population assessment of some of the kinematic features shown for individual subjects in Figure 11–12. Overall, these analyses pointed out a dependence of the mouse kinematics on the initial segment of the ball trajectory, but also a remarkable degree of variability and idiosyncrasy. For example, in Experiment 1, movements were initiated at significantly different latencies in response to unperturbed 1 g trajectories that followed either a short or a long path because of the different launch angles (F_(17,170)_ = 11.7, p<0.001). Subjects started the mouse movement later for long 1 g trajectories (experimental conditions 7,9,11 in Figure 13) than for short 1 g trajectories. Instead, in Experiment 2, where ball trajectories had the same launch angle, movement onsets did not depend significantly on the ball trajectory. There was also a fair degree of idiosyncrasy among subjects. In Experiment 1, Group 1 subjects showed significantly later movement onsets compared to Group 2 subjects (F_(1,10)_ = 26.2, p<0.001), whereas no significant differences were observed, in Experiment 2, between Group 3 and Group 4 subjects. The latency of the maximal peak velocity was also significantly different among ball trajectories in both Experiment 1 (F_(17,170)_ = 15.3, p = 0.03) and Experiment 2 (F_(17,170)_ = 10.6, p<0.001). However, these effects had different basis in the two experiments. In Experiment 1, the latencies of the maximal velocity peak paralleled the trend observed for the movement onset by varying in response to 1 g trajectories with different launch angles. Instead, in Experiment 2, maximal velocity latencies differed between 2 g trajectories with either short or long perturbation intervals. The maximal peak of mouse velocity occurred significantly earlier with occluded than entirely visible trajectories (F_(17,170)_ = 6.07, p<0.001), and had also significantly different latencies between the two groups of subjects (F_(1,10)_ = 9.6, p = 0.011). However, these latter two effects were observed only in Experiment 1, suggesting that they might reflect idiosyncratic behavior among subject groups. Consistent with the results reported for the maximal peak latencies, maximal mouse speed depended similarly on the ball trajectory (F_(17,170)_ = 20.7, p<0.001 for Experiment 1; F_(17,170)_ = 6.4, p<0.001 for Experiment 2).

Movement durations were significantly longer during 0 g than 1 g and 2 g trajectories, reflecting the longer duration of 0 g trajectories compared to 1 g and 2 g trajectories (F_(17,170)_ = 107.8, p<0.001 and F_(17,170)_ = 132.3, p<0.001 for Experiment 1 and Experiment 2, respectively). Mouse movements were also significantly shorter with occluded trajectories (F_(1,10)_ = 18.7, p = 0.001 for Experiment 1; F_(1,10)_ = 10.01, p = 0.01 for Experiment 2), particularly during perturbed 0 g trajectories (interaction between ball trajectory and session type: F_(17,170)_ = 6.4, p<0.001 and F_(17,170)_ = 6.1, p<0.001 for Experiment 1 and Experiment 2, respectively). Finally, in Experiment 1, movement durations differed between Group 1 and Group 2 subjects (F_(1,10)_ = 27.4, p<0.001), mirroring the effects on the movement onset and on the latency of the maximal velocity peak.

**Figure 14:**
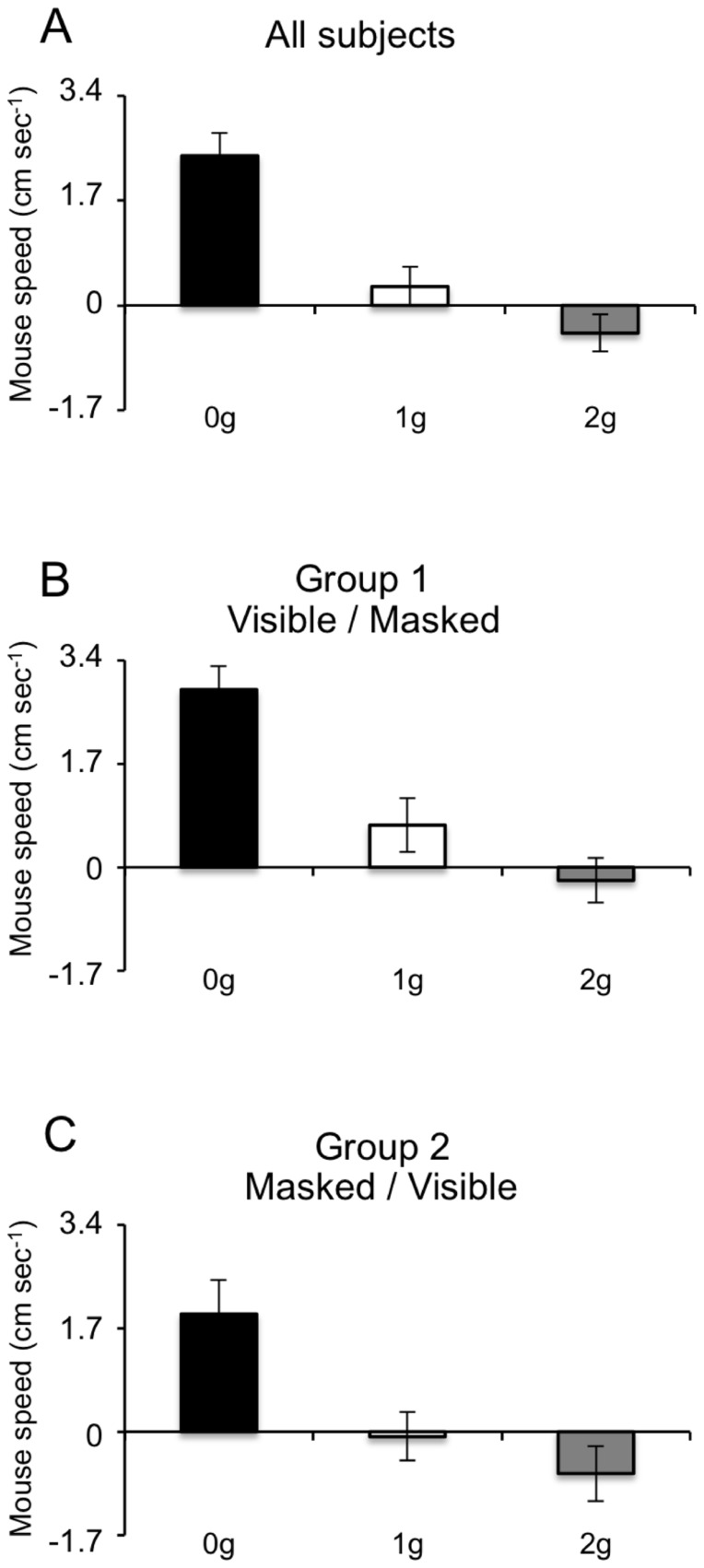
Mouse velocity at the button press. Mouse velocities recorded during the Visible session of Experiment 1 and plotted for all (A), Group 1 (B) and Group 2 (C) subjects. Bars represent mean values (± SEM) computed across subjects and across trials with the same law of motion. Black, white and grey bars represent 0 g, 1 g and 2 g trials, respectively.

The last two indexes namely, the velocity at the time of the button press and the direction of the last mouse sub-movement were sampled after the trajectory perturbation and, thus, could be related to the ball acceleration, to the session type and to the session order by means of multivariate regression models. The results of these analyses were congruent with those of the button press responses. For example, the mouse velocity at the button press depended significantly on the ball acceleration (F_(2,422)_ = 368.7, p<0.001), with 0 g trials showing large positive (rightward) velocities, 1 g trials smaller positive or nearly null velocities and 2 g trials, on average, negative (leftward) values (Figure 14). This result may be consistent with initial estimates of the ball trajectories based on the effect of gravity, which were corrected by rightward adjustments of the mouse position during 0 g trajectories and by leftward adjustments during 2 g trajectories. In fact, this monotonic trend was significantly stronger when trajectories were entirely visible (main effect of session type: F_(1,422)_ = 12.0, p<0.001; interaction between ball acceleration and session type: F_(2,422)_ = 45.9, p<0.001), suggesting that adjustments of the mouse cursor position were mainly based on visual feedback. Interestingly, Group1 and Group 2 subjects showed significantly different trends (main effect of session order: F_(1,422)_ = 80.0, p<0.001; interaction between ball acceleration and session order: F_(2,422)_ = 10.9, p<0.001). Group 1 subjects showed positive velocities for 0 g and 1 g trials and slightly negative velocities for 2 g trials, whereas Group 2 subjects showed positive velocities for 0 g trials, nearly zero for 1 g trials and negative for 2 g trials. Thus, in agreement with the results of the button press responses, Group 2 subjects' behavior appeared more congruent with expectation of the effect of gravity on the ball trajectories. Analysis of the direction of the last mouse sub-movement supported further these observations. The fraction of rightward (positive) velocity peaks decreased monotonically from 0 g to 2 g trials (Z = 2.5, P = 0.014) and this trend depended significantly on the session order (Z = 2.9, P = 0.004). In fact, a higher percentage of leftward (negative) velocity peaks was observed in 2 g trials of Group 2 subjects, in accord with the negative velocities at button press reported in this group of subjects.

## Discussion

This paper investigated the manual interception responses to simulated fly-ball trajectories that could be perturbed with laws of motion incongruent with natural gravity and occluded for variable periods of time. The combination of three laws of motion, three visual occlusion intervals and different initial exposures to the altered trajectories, produced potentially interesting findings on the issue of how real-time and pre-conceived information about object motion may be integrated in the control of the interceptive action. Insights on the relative contribution of feedback processes based on real-time visual signals and a-priori information emerged, first, by examining the interceptive responses during the Visible session compared to the Masked session. Second, the changes in the interceptive responses observed by varying the length of the occlusion interval and the initial exposure to the perturbed trajectories provided indication on the putative nature of the predictive processes underlying the visual extrapolation and the manual interception of the occluded targets.

When vision was available throughout the ball trajectory, a coexistence of predictive and feedback-based control for the interception of the target motion, of the kind reported recently by Katsumata and Russell [87], was evident from both button press responses and mouse movements. The systematic temporal underestimate of 0 g trajectories observed consistently across subject groups and the two experiments, for example, could represent evidence in favor of predictive estimate of the ball motion. This finding is, in fact, congruent with results of previous studies that manipulated the law of motion of visual targets in vertical free-fall and found similar temporal underestimate of constant velocity and decelerated targets [49]–[50], [80], supporting the idea that subjects expect the effects of Earth's gravity even in cases of gross distortions of the simulated law of motion. Expectation of gravity effects was also evident from the mouse movement kinematics, particularly, from the distributions of mouse cursor velocities at the button press. In line with this interpretation, mouse velocities were either very low or almost null for 1 g trajectories, whereas for perturbed trials they were higher and had opposite directions, reflecting under- and overestimate of 0 g and 2 g trajectories, respectively. Note that the systematic overestimate of 2 g trajectories predicted by the internal model of gravity was also remarked by a higher number of leftward mouse sub-movements preceding the button press made by subjects during 2 g compared to 0 g and 1 g trials. These corrective mouse sub-movements minimized effectively the position error (see for example, 0 g trials in Experiment 2), suggesting that on-line visual feedback mechanisms were integrated with ball trajectory predictions to achieve precise mouse positioning. The potential contribution of feedback mechanisms was underlined further by the scaling of the mouse movement duration with the ball trajectory duration, which may imply velocity coupling between the hand and the target [9], [11], [14], [18], [36], and by the significant dependence of the movement onset and of the maximal peak velocity on the kinematics of the initial segment of ball trajectory. Subjects, however, did not seem to adopt a continuous visual feedback strategy like proposed for ball catching in the baseball field [19]. They used information about the initial portion of the trajectory to predict where the ball may land and reached the interception area with the mouse cursor much in advance relative to the ball. Then, final corrections of the position of the mouse cursor were made right before the button press response (see above). Field measurements and virtual reality manipulations have suggested, instead, a continuous servo-control strategy, known as optic acceleration cancellation, to explain the ability of fielders to intercept fly-balls [19]–[22], [88]–[90]. According to this theory, experienced athletes may achieve the correct position for ball catching by running at a speed such that the tangent of the angle of gaze from the fielder to ball increases at a constant rate. Although the optical cancellation strategy has been shown to account for the interceptive behavior of fielders across a fair range of fly-ball trajectories, numerical simulations have implied that it may not represent a unique interceptive strategy for catching fly-balls, because it does not generalize across a wider range of possible fly-ball trajectories and fielders' behavior [91]. The results of these simulations raised, in fact, the possibility that a mixture of feedback and predictive mechanisms, like the one observed in the present study, may afford a wider generalization of the interceptive behavior [91]. Aside from these considerations, it must be remarked that the different environmental context could represent a major source of discrepancy between the interceptive strategies observed in our computer virtualization of the baseball game and in the real baseball. In the real game situation, motion of the ball in depth generates significant looming when approaching the baseball player and the interceptive action involves running towards the ball landing area and catching the ball with a quick arm extension and finger closure, having both visual and haptic feedback of the catch. Here, instead, ball motion was tangential to the observer and, therefore, did not generate looming information. The interceptive action involved displacing an external device to control the motion of an actor on the visual display and a button press to signal the catch, with only visual feedback of the ball interception. In this respect, our experimental situation is similar to that of Saxberg's [92]. This study, in fact, employed a 3D video game in which subjects, by moving a mouse, displaced a triangular-shaped object into a position where a simulated projectile would land. Interceptive ability in this task could not be accounted for by Chapman's servo-control strategy [19], but relied heavily on looming information and it was consistent with a predictive strategy [93]. Thus, differences in the visual input, as well as in the nature of the motor action required to intercept the target, could partly account for the different interceptive strategy observed in the present task compared to the real baseball situation.

While with entirely visible ball trajectories predictive and prospective control of the interceptive action coexisted, when vision of the target was occluded before the interception, predictive mechanisms became predominant, as indicated by the pattern of interceptive errors observed during both experiments in response to occluded non-natural projectile trajectories.

Consistent with predictive spatial and temporal estimates based on expectation of Earth's gravity effects on the ball motion, 0 g trajectories were temporally and spatially underestimated, whereas 2 g trajectories were overestimated. This result extends the earlier finding that, for relatively short occlusion intervals, interception timing reflected an internal model of gravity [80]. Here, in fact, we provided similar evidence by using much longer occlusion intervals and also with respect to the spatial estimates of the interceptive response, which had not been taken into account in the earlier study. An alternative hypothesis proposed to explain a spatial bias in interceptive actions involving lateral hand movements suggests that it may reflect incomplete extrapolation of the occluded targets due to reduction of the visuomotor gain, i.e. the relationship between the hand and the target velocity [77]. With respect to the present experimental data, this hypothesis would predict a systematic leftward bias of the mouse cursor position, larger for accelerated motion than for 0 g, constant velocity, trajectories. However, mouse position errors did not comply with this prediction. As shown by Table 2 and Figures 6–7, leftward biases, denoting ball trajectory underestimates, were not found consistently across ball trajectories and they were always larger for 0 g than accelerated trajectories. Moreover, the results of Experiment 1, where 1 g trajectories were matched for spatial length to the perturbed 0 g and 2 g trajectories, indicate that positional errors depended, in effect, on the ball law of motion rather than on the spatial length of the ball trajectory, as observed originally in the study of Dessing et al. [77]. Finally, position and timing errors showed congruent bias directions across ball accelerations (i.e. 0 g underestimate and 2 g overestimate occurred both in the temporal and spatial domains), reinforcing the idea that they reflected estimates of the ball trajectory based on implicit knowledge of gravity.

In agreement with the conclusion previously reached by Baurès and Hecht [94], prediction of the ball motion based on the expectation of gravity effects became an increasingly stronger factor as the availability of visual motion information was progressively reduced. Temporal and position errors, in fact, increased significantly as a function of the occlusion interval, but with larger increases for 0 g and 2 g perturbed trials than for unperturbed 1 g trials, which underwent minimal changes. This finding could be explained parsimoniously by assuming contribution from at least two types of information for the extrapolation of the occluded motion: short-term visual memory signals derived from the information available before ball disappearance [67]–[74] and signals related to a-priori knowledge of gravity effects on the ball motion [53]–[56]. However, short-term visual memory signals are known to decay exponentially with time [95]–[97], with differential impact on the spatial/temporal estimates of the perturbed trajectories compared to those of 1 g trajectories, which may rely more on a-priori knowledge of gravity.

Multiplex interaction of short and long-term information for the extrapolation of occluded motion emerged also from the different patterns of interceptive errors observed between subjects without previous visual experience of the perturbed trajectories and subjects that acquired visual experience of the ball trajectories by performing first the Visible session. The former subjects (Groups 2 and 4) intercepted the occluded targets by seemingly accounting for the effects of gravity, since their 1 g responses were closest to correct, 0 g responses were anticipated and spatially underestimated, and 2 g responses were late and overestimated. Instead, Group 1 and Group 3 subjects, who first experienced perturbed and unperturbed trajectories in full vision (without occlusion), showed early and spatially underestimated responses to 0 g trials, but rather close spatial and temporal responses to accelerated 1 g and 2 g trials, suggesting that knowledge of the visual properties of the altered trajectories was combined with information related to the internal model of gravity. Therefore, the extent that spatial/temporal estimates of the occluded motion were based on a-priori knowledge of gravity depended critically on prior visual experience of the altered trajectories. Conversely, the possibility that apparent effects of session order could be surrogated by inter-group variability was ruled out clearly by two accounts. First, group differences related to the session order were consistent between the two experiments, making it unlikely that they occurred by chance; second, in spite of the overall different distributions of TE and PE across ball accelerations, subject groups 2 and 4 showed responses to 0 g trials that were hardly distinguishable from those observed in Groups 1 and 3, meaning that interceptive responses, in general, were not idiosyncratic to subject groups. The notion that both a-priori knowledge of gravity and prior visual experience of the altered trajectories contributed to predictive estimates of the ball trajectory was also supported, in Experiment 1, by the significant effects of the session order on the mouse velocities at button press and on the direction of the last movement subcomponents observed among ball accelerations. These effects denoted again an interceptive behavior more congruent with spatial predictions based on presupposed knowledge of gravity for Group 2 compared to Group 1 subjects.

Overall, these results imply that internal representations of the altered visual trajectories were developed during the Visible session and retrieved about 30 days later during the Masked session. Recent observations that practice with arbitrary accelerations favors the development of novel internal models for the interception of occluded trajectories support, in effect, this possibility [81]. In fact, adaptation of interceptive responses to the perturbed trajectories occurred for both 0 g and 2 g trials with rather fast time-constants, similar to those reported previously for the interception of constant velocity targets in vertical free fall [50], [98]. As also reported by Zago et al. [50], the adaptation was not complete because the interception error at the learning asymptote remained above the threshold considered for a successful response. It is likely that the rather small error tolerance for an interceptive response to be considered successful made the task too difficult, limiting the ability of subjects to improve their overall performance. The rapid, but limited, adaptation of the interceptive responses might also suggest that novel visual representations of the perturbed trajectories were based on relatively simple adaptive neural mechanisms. Perceptual priming, for example, is a rather fast and simple form of implicit memory, driven by repeated visual stimuli and mediated by plastic changes in cortical visual areas [99]–[101]. In particular, repeated visual motion stimuli are known to induce perceptual priming in visual motion area hMT/V5+ [102]–[103] and priming effects have been reported recently for interceptive actions [104], suggesting that this mechanism may reasonably account also for our observations. Another element in favor of this interpretation is that learning occurred also without response feedback (see Experiment 1), being driven mainly by the repeated presentation of the perturbed motion. Learning novel visual representations of the perturbed trajectories could also interfere with pre-existing information related to the internal model of gravity through a mechanism known as forgetting (see [105] for a review). This was evident in the behavior of Group 4 subjects, who intercepted the occluded trajectories during the first experimental session by relying mainly on estimates based on an internal model of gravity (see Figure 9). However, about thirty days later during the Visible session, they showed a behavior compatible with partial forgetting of the 1 g internal representation, indicated by the exponential increase of the interception error for the unperturbed 1 g trials, which paralleled the exponential decrease for perturbed 0 g and 2 g trials.

Finally, another implication of the present findings is that spatial and timing aspects of the interceptive action may be controlled in parallel. Regardless of whether the trajectories were fully visible or not, the final position of the mouse cursor for target interception was, in fact, specified much in advance relative to the button press, which indicated the time of the interceptive event. Clinical and neurophysiological evidence that spatial location and timing of visual stimuli are processed along independent temporo-parietal pathways may be in line with this view [106]–[107]. Moreover, recent fMRI results have indicated that spatial and temporal extrapolation tasks activate different sets of brain areas, even though with fair degree of overlap [69]–[70]. The distribution of both spatial and temporal errors in the present study, however, followed rather similar trends across experimental conditions, implying that the neural processes that contribute to the representation of the target kinematics might occur either upstream or in overlapping regions of the networks that the control the spatial and temporal aspects of the interceptive action.

In conclusion, the results of the present study indicate that, at least under our experimental conditions, predictive and feedback mechanisms coexist for the control of the interceptive action when vision of the target is available throughout its trajectory. Instead, when vision of the target is occluded for relatively long time intervals, a-priori knowledge of the visual environment becomes a major contributor to the visual extrapolation process and, thus, to the target interception. An internal model of the effects of natural Earth's gravity was found to be the major factor accounting for the spatial and temporal estimates of the target interception, but also knowledge of features that are not congruent with a natural environment, acquired through previous visual experience, may contribute significantly.
